# Efficient Regeneration of Graphite from Spent Lithium-Ion Batteries through Combination of Thermal and Wet Metallurgical Approaches

**DOI:** 10.3390/ma17163883

**Published:** 2024-08-06

**Authors:** Riquan Yu, Changyou Zhou, Xiangyang Zhou, Juan Yang, Jingjing Tang, Yaguang Zhang

**Affiliations:** School of Metallurgy and Environment, Central South University, Changsha 410083, China

**Keywords:** spent lithium-ion batteries, anode materials, graphite, electrochemical performance, regeneration

## Abstract

With the large-scale application of lithium-ion batteries (LIBs) in various fields, spent LIBs are considered one of the most important secondary resources. Few studies have focused on recycling anode materials despite their high value. Herein, a new efficient recycling and regeneration method of spent anode materials through the combination of thermal and wet metallurgical approaches and restored graphite performance is presented. Using this method, the lithium recycling ratio from spent anode materials reaches 87%, with no metal impurities detected in the leaching solution. The initial Coulombic efficiency of the recycled graphite (RG) materials is 90.5%, with a reversible capacity of 350.2 mAh/g. Moreover, RG shows better rate performance than commercial graphite. The proposed method is simple and efficient and does not involve toxic substances. Thus, it has high economic value and application potential in graphite recycling from spent LIBs.

## 1. Introduction

The demand for lithium-ion batteries (LIBs) has increased with the rapid development of electronic products and electric vehicles because of their high energy density, excellent rate performance and good cycle performance [1,2,3]. According to statistics, the global LIB sales in 2023 were 1202.6 GWh, and the global LIB demand could reach 3600 GWh by 2030 [4,5,6]. The average service life of LIBs is 3–5 years, and the service life of vehicle batteries is approximately 5–8 years [7,8]. The global production of spent LIBs is estimated to exceed 3.7 million tonnes by 2030 [9,10]. Currently, most studies on recycling waste LIBs focus on cathode recycling, with inadequate research on anode materials, which are treated as solid wastes in the recycling process, because of the difference in the value of these materials [11,12,13,14]. Analysis of the anode materials of waste LIBs revealed that the lithium content in the used anode materials is approximately 30 mg/g, far exceeding that found in lithium ore. Thus, it can be a huge lithium resource.

Moreover, the structure of anode materials hardly changes, and the graphite layered structure remains intact [15,16]. Thus, high-value recycling of anode materials of waste LIBs must be investigated. Chen J.P. [11] designed a small-scale production line for the green recycling of used battery materials. They washed the used battery materials with NaOH and ball-milled them to obtain the recycled cathode and anode materials. However, the recycling process requires multiple ball-milling rounds, making it long and complicated. Detailed experiments and tests of anode material recycling have not been provided. Zhang J [17] used phenolic resin as a carbon source to coat the surface of the waste anode materials with amorphous carbon to restore its electrochemical performance. The performance of the treated materials was improved. Nevertheless, recycling the lithium in the anode was not considered and the regeneration process was complicated and expensive because of the coating cost, making it unsuitable for practical production applications.

In this study, a lithium recycling method for spent anodes has been proposed, and the recovery of the electrochemical performance of the anode materials has been carefully investigated. The powder of spent LIB anodes provided by a factory has been used as the raw material in the experiment. Three simple steps combined with thermal and wet metallurgical approaches have been conducted in this study: (1) heat treatment of the spent anode materials under an inert atmosphere to remove the organic components such as binder and separator debris, (2) stirring of the heat-treated materials with deionized water followed by filtering to allow most of the lithium in the materials to leach selectively and (3) washing the stirred and filtered materials in nitric acid to remove the metal impurities remaining in the materials. The results show that the electrochemical performance of the treated anode materials has recovered, with their main indicators reaching those of commercial graphite (CG). Thus, they can be directly reused as battery-grade anode materials. The lithium recycling ratio in the waste anode materials has reached 87%, and other metal impurities have not been detected in the leaching solution. Thus, the materials can be recycled without further steps to remove impurities. This simple and efficient method generates no toxic substances, and all of the components of the anode materials are recycled with high recovery rates. Thus, this method is highly suitable for practical applications.

## 2. Materials and Methods

### 2.1. Materials and Reagents

Anode powder of waste LiFePO_4_ LIBs was provided by Hunan Chenyu Fuji new energy Technology Co., Ltd. in Changde, China, which was used as the raw material in the experiment. The solutions used in the experiment were all prepared using deionized water, and all of the reagents were of analytical grade.

The metal mass content of the above spent anode powder was determined by completely dissolving the metals using aqua regia and testing their amounts in the solution. The solid/liquid ratio was 10 g/L, and the mixture was stirred for 6 h at 60 °C. Cu and Li were detected in the solution, and after three parallel experiments were performed, the average values of their mass contents were calculated as 2.95% and 6.54%, respectively.

### 2.2. Lithium and Graphite Recovery

**Figure 1:**
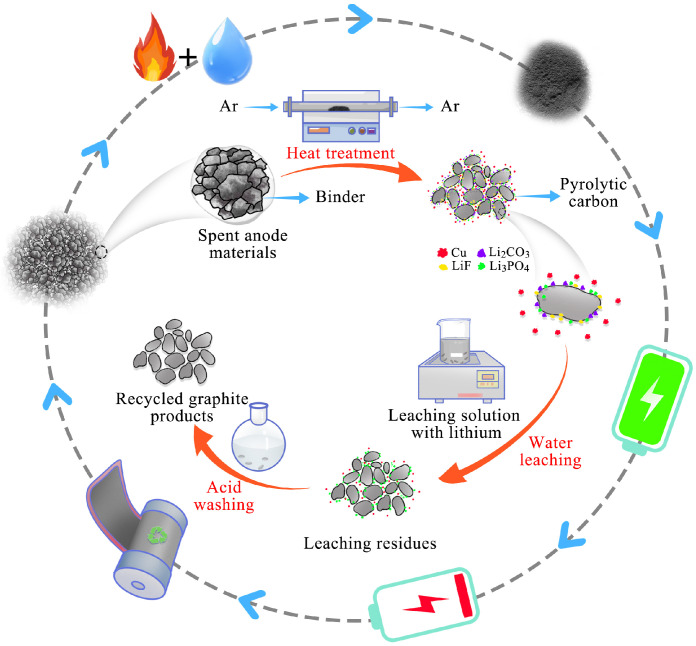
Schematic illustration of recycling process of spent anode materials.

Three key steps were conducted in this recycling process to recover the spent anode materials. Figure 1 shows a flow chart of the recycling process of the spent anode materials.

In the first step, organic components, such as the binder and separator debris, in the spent anode materials were removed. This was achieved by placing the spent anode materials in a tube furnace under an inert atmosphere (i.e., highly pure argon), which was injected into the furnace. The temperature was increased to 600–800 °C at a rate of 5 °C/min and maintained for 1 h. In the second step, the lithium in the anode materials was mostly water-soluble. The heat-treated spent anode powder was placed in a beaker with a certain amount of deionized water for stirring to recycle this part of the lithium. The leaching process was performed in a thermostatic water bath with magnetic stirring. After the reaction, solid–liquid separation was conducted using a vacuum filter to obtain the leachate containing Li^+^ and the anode materials to be treated. The effects of time, temperature, and the solid/liquid ratio on the leaching process were studied. In the third step, the impurities remaining in the materials, which considerably affect their electrochemical performance, were removed by placing the materials in a beaker and adding nitric acid (concentration = 4 mol/L) at a solid/liquid ratio of 20 g/L. Next, the solution was stirred for 2 h at 60 °C. Recycled graphite (RG) was then filtered, washed and dried.

### 2.3. Characterisation

A simultaneous thermal analyser (SDT650) measured the thermal stability of the materials. X-ray diffraction (XRD, Rigaku-TTRIII, Rigaku, Japan) was used to determine the materials’ phase composition and crystal structure using Cu-K as a radiation source within a scanning range of 10–80° at a scanning rate of 10°/min. Scanning electron microscopy (SEM, Nova NanoSEM230, FEI, Rock Hill, SC, USA) and energy dispersive X-ray spectroscopy (EDS, Thermo Fisher, Waltham, MA, USA) were used to observe the surface morphology and composition of the materials, respectively. The specific surface area parameters of the materials were measured using a specific surface area analyser (BET, QUADRASORB evo, Anton Paar, Austria). An inductively coupled plasma optical emission spectrometer (ICP–OES, ICAP7400Radial, Thermo Fisher, Waltham, MA, USA) measured the metal element concentrations in the solution.

### 2.4. Electrochemical Test

RG, an acetylene black conductive agent, a binder (LA-133) and a carboxymethyl cellulose dispersant (the above three materials were bought from DoDo Chem, Suzhou, China) were uniformly mixed at a ratio of 90:5:3:2, using deionized water as a solvent. The materials were uniformly ground, coated on the copper foil and placed in a vacuum drying oven at 100 °C for 12 h to dry. The dried electrode pieces were pressed to obtain the working electrodes. A CR2025 coin-type half-cell was fabricated in a glove box under a protective argon atmosphere with metallic lithium, a polypropylene film and a lithium hexafluorophosphate solution in ethylene carbonate, dimethyl carbonate and diethyl carbonate (LiPF_6_-EC/DEC/DMC, 1 M) as the counter electrode, separator and electrolyte, respectively. A LAND CT2001A battery tester (LAND instruments, Wuhan, China) was used for constant current charge–discharge testing within a voltage range of 0.01–2 V and a current density of 0.2 C (1 C = 372 mA/g). Cyclic voltammetry (CV) was performed using an electrochemical workstation (PARSTAT 4000, Princeton, NJ, USA) at a scanning speed of 0.1 mV/S and a scanning voltage range of 0.01–2.5 V. Electrochemical impedance spectroscopy (EIS) was performed using an electrochemical workstation at a test frequency range of 100–10 mHz. All electrochemical tests were performed at 25 °C.

## 3. Results and Discussion

### 3.1. Heat Treatment

**Figure 2:**
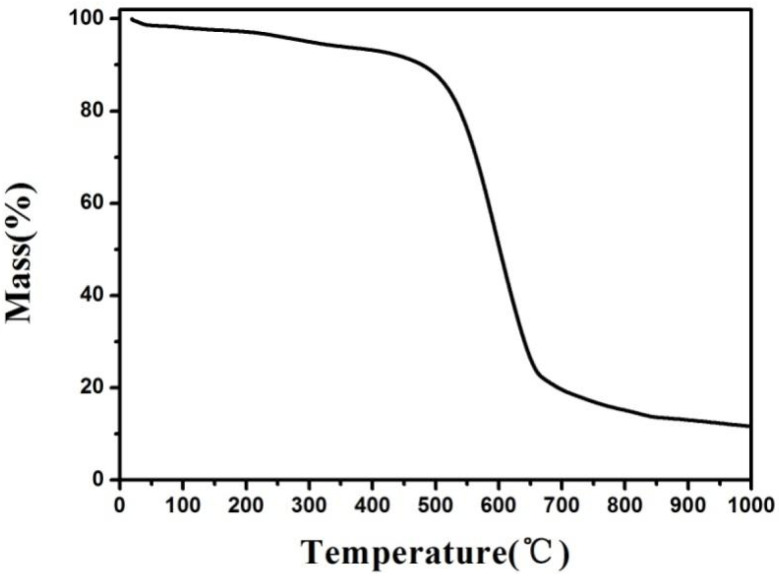
The thermogravimetric curve of the spent anode materials under an air atmosphere.

Thermogravimetric analysis determined the mass change of the spent anode materials with the increased temperature in an air atmosphere. Figure 2 shows that the mass loss ratio of the spent anode materials considerably increased at 500 °C, reaching its maximum at 580 °C. At 700 °C, the materials were almost completely oxidized and the remaining materials were mainly impurities remaining in the waste anode materials. Because the temperature in this study reached 800 °C, the heat treatment experiments were performed under a highly pure argon protective atmosphere. 

**Figure 3:**
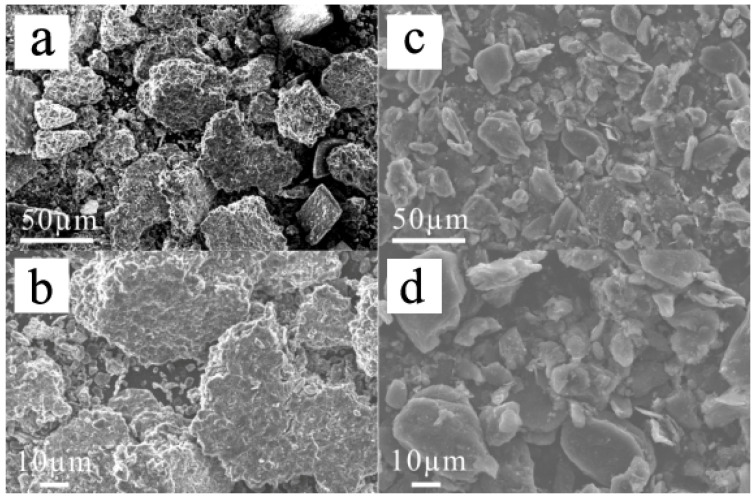
SEM images of the heat-treated anode materials: (**a**,**b**) RAW; (**c**,**d**) heat treatment at 800 °C.

The SEM results (Figure 3) show the surface morphology of the materials. The figure shows that the graphite particles were not smooth after a long period of use and there were many sticky substances on their surface. The overall particle size increased and the particle size was unevenly distributed over the material. The composition of the negative electrode indicated that the viscous substances comprised the residual electrolyte, solid–electrolyte interface (SEI) film and binder. After the heat treatment of the spent anode materials under a protective inert atmosphere, high-temperature carbonisation of the viscous substance resulted in covering the particle surface with amorphous carbon. After removing the impurities, such as the binder, residual electrolyte and organic SEI components, using high temperature, the particle size of the materials considerably decreased, showing a more uniform distribution.

Table 1 indicates that after the heat treatment, the specific surface area of the waste anode materials decreased from 7.059 to less than 4 m^2^/g. The specific surface area gradually decreased with the increase in the heat treatment temperature. After the heat treatment at 800 °C, the specific surface area decreased to 3.1 m^2^/g, indicating that the surface impurities were gradually removed with the increase in the heat treatment temperature. According to GB/T24533-2009 [18], the specific surface area of artificial graphite is approximately 1.5 m^2^/g. An excessively high specific surface area will reduce the initial Coulombic efficiency due to more SEI film produced. Although the specific surface area was optimized after the heat treatment, it should be further reduced. 

**Figure 4:**
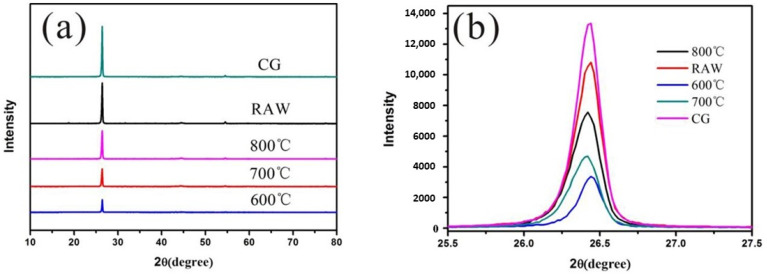
(**a**) XRD patterns of spent anode materials and recycled graphite products at different heat treatment temperatures; (**b**) enlargement curves around 26.5°.

XRD was used to analyse the spent anode materials and RG at different heat treatment temperatures (Figure 4). The spent anode materials exhibited a strong diffraction peak around 26.5°, which is a characteristic graphite peak related to (002) lattice planes. After a long period of use, the graphite structure did not show a considerable change, maintaining a complete graphite layered structure with good crystallinity. However, owing to the high content of impurities in the spent anode materials and the binder coating on their surface, the peak height of the spent anode materials was lower than that of CG. After the heat treatment of the spent anode materials, their peak intensity decreased slightly because of the carbonisation of the impurities, such as organic binders and separator debris, in the materials into amorphous carbon owing to the high temperature, forming a composite material of graphite and amorphous carbon, which reduced the peak intensity. Figure 4b shows that the peak height of the spent anode materials after the heat treatment gradually increased with the heat treatment temperature, indicating a decrease in the content of impurities and a gradual increase in the degree of crystallinity. Therefore, 800 °C is the optimal heat treatment temperature.

### 3.2. Selective Leaching of Llithium

**Figure 5:**
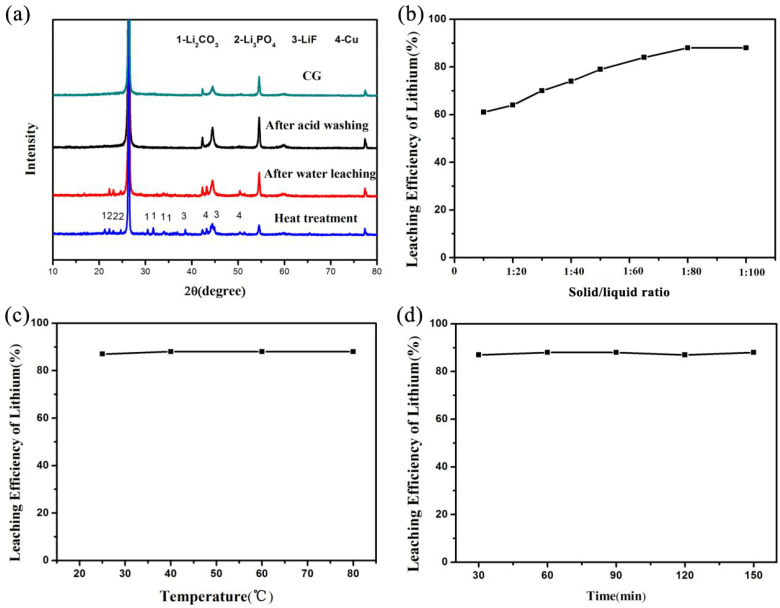
(**a**) XRD patterns of spent anode materials after different processing steps. Effects of (**b**) solid/liquid ratio, (**c**) temperature and (**d**) time on leaching efficiency of lithium.

After the heat treatment of the waste anode materials, the binders covering the surface of the materials were carbonised, facilitating the dissolution of the lithium in the materials. Figure 5a shows that after the heat treatment of the spent anode materials, lithium was mainly present as Li_2_CO_3_, Li_3_PO_4_ and LiF, Cu was also presented in the mixture. The lithium content in the spent anode materials was approximately 3 wt%, showing a high recycling value. Deionized water was used as the leaching reagent to achieve selective lithium extraction.

#### 3.2.1. Effect of Solid/Liquid Ratio on Leaching

The effects of different solid/liquid ratios (1:1–1:100) on the leaching process were investigated at a leaching temperature of 60 °C and a leaching time of 2 h. The experimental results are shown in Figure 5b. The figure shows that changing the leaching solid/liquid ratio affected the leaching process considerably. Lithium leaching increased with the increase in the solid/liquid ratio. At 1:10, the lithium leaching ratio was approximately 61%, reaching 88% at 1:80. However, increasing the solid/liquid ratio to values higher than 1:80 did not significantly affect lithium leaching. Because lithium is water-soluble to some extent, the lithium in the spent anode materials gradually dissolved with the increase in the solid/liquid ratio because although its theoretical solubility is not high, its content in the anode materials was relatively low, allowing it to be completely dissolved by increasing the added amount of deionized water. Figure 5a shows that after extracting the lithium by water, the peaks of Li_2_CO_3_ and LiF in the anode materials disappeared, indicating that they were removed in water. Thus, the solid/liquid ratio during leaching should be 1:80.

#### 3.2.2. Effect of Temperature on Leaching

The effect of different temperatures (25–80 °C) on the leaching process was explored at a solid/liquid ratio of 1:80 and a leaching time of 2 h. Figure 5c shows the experimental results. Changing the leaching temperature exhibited little effect on the leaching process. With the increasing temperature, the leaching ratio of lithium hardly changed. At 25 °C, the leaching ratio reached 87%. Although solubility mostly increases with temperature, the amount of water added was far greater than the lithium content in the materials. Thus, the lithium solubility at low temperatures was sufficient to achieve full leaching.

Similarly, the solubility of Li_2_CO_3_ at high temperatures is lower than that at normal temperatures. However, in this case, its solubility at normal temperatures meets the minimum requirements for complete dissolution. Thus, the temperature exhibited a minimal effect on the lithium leaching ratio. Therefore, the optimal leaching temperature is 25 °C.

#### 3.2.3. Effect of Time on Leaching

The effects of different leaching times (30–150 min) on the leaching process were explored at a solid/liquid ratio of 1:80 and a leaching temperature of 25 °C. Figure 5d shows the experimental results. Changing the leaching time did not strongly affect the leaching process. After 30 min, the leaching ratio of lithium reached 87%. However, with the increase in the leaching time, the leaching ratio of lithium hardly changed. The water-soluble lithium salt in the anode materials exhibited a high dissolution rate. It was completely dissolved after 30 min, indicating that water extraction of lithium is very efficient and can be used for practical applications. Thus, the optimal leaching time of lithium is 30 min.

### 3.3. Acid Washing for Impurity Removal

After the first charge–discharge cycle of LIBs, the anode materials and electrolytes react at the solid–liquid interface, and an SEI film forms on the surface of the anode materials. The formation of the SEI film is critical to the performance of the anode materials. Some lithium salt and metal impurities remain on the surface of the spent anode materials obtained after water extraction of lithium, resulting in uneven surface morphology of the materials, which increases the thickness and unevenness of the SEI layer during the first charge–discharge cycle. The SEI layer is more likely to rupture during the battery cycling process, leading to further consumption of the lithium in the battery, which reduces the Coulombic efficiency and cycling performance [19,20]. Thus, nitric acid was used as the leaching reagent to remove the metal impurities, such as copper and Li_3_PO_4_, remaining on the surface of graphite after water leaching.

**Figure 6:**
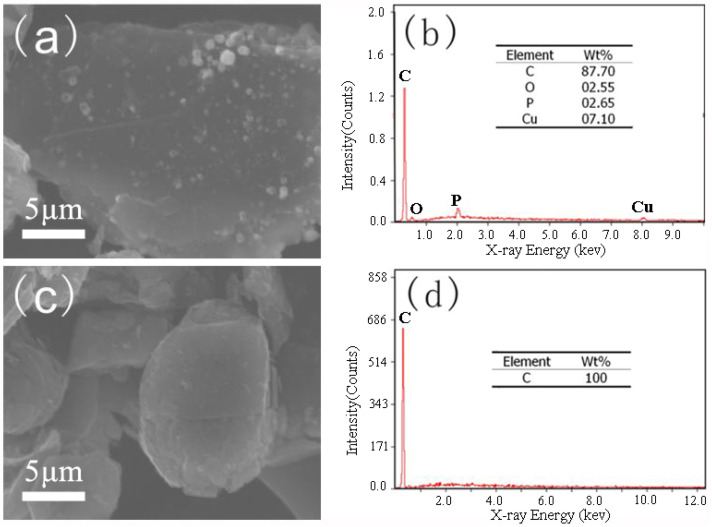
SEM images of anode materials before acid washing (**a**); anode materials after acid washing (**c**); corresponding EDX spectra of anode materials before acid washing (**b**) and anode materials after acid washing (**d**).

Figure 6a shows that the surface of the spent anode materials after the heat treatment was not smooth and was covered by impurities, making it unsuitable for SEI layer formation. The EDS diagram indicates that the materials contained several elements, such as P, O and Cu, in addition to C. Most impurities were Cu particles with some P and O, which were mainly from Li_3_PO_4_ and could not be eluted in the water leaching step.

After the materials were acid-washed to remove impurities, all impurity particles on the waste anode materials’ surface disappeared and the materials exhibited a smooth surface morphology. According to the EDS diagram, only C was detected in the materials, and the other elements were undetectable because their contents were lower than the detection limit. Figure 6d shows only the graphite peak and all impurity peaks disappeared, confirming the strong impurity removal ability of acid washing. Table 1 indicates that the specific surface area of the materials after acid washing (i.e., RG) decreased from 3.199 to 2.027 m^2^/g, which is very close to that (1.505 m^2^/g) of CG. This can be attributable to the removal of impurity particles from the material surface and the smoother surface morphology of the materials after acid washing. 

### 3.4. Electrochemical Performance

**Figure 7:**
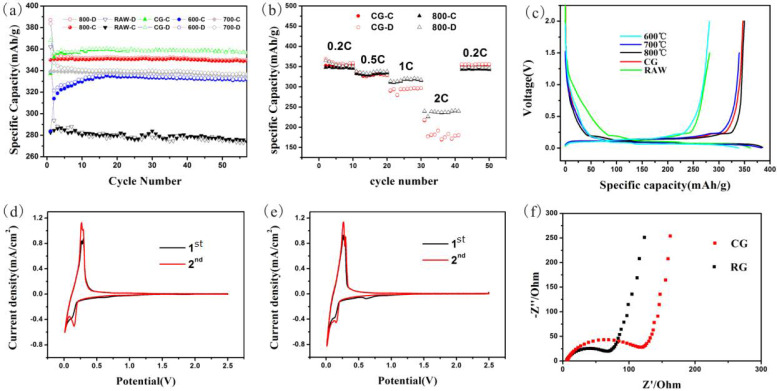
(**a**) The cycle performance of the recycled anode materials at a current density of 74.4 mA/g (-C = charge, -D = discharge); (**b**) the rate performance of CG and RG; (**c**) the initial charge and discharge of the recycled anode materials; the CV curves of (**d**) CG and (**e**) RG; (**f**) EIS of CG and RG.

The recycled anode materials obtained through the proposed process were used to construct a half-cell for electrochemical testing, and the results are shown in Figure 7. In this figure, the spent anode materials treated at the optimal heat treatment temperature (800 °C) were considered RG, and Table 2 summarises the data on the electrochemical performance of different samples.

Figure 7 shows that the initial Coulombic efficiency and capacity of the untreated raw materials were far lower than those of CG. Moreover, the materials exhibited poor cycle stability with a declining capacity compared to their initial value. This is due to the increased impurity content in the spent anode materials, which deteriorates the surface morphology and increases the specific surface area, leading to unsuitable conditions for SEI layer formation and difficulty in the insertion and extraction of lithium ions.

After the proposed treatment process, the capacity of the recycled anode materials and their initial Coulombic efficiency largely improved compared to the untreated raw materials. With the increase in the heat treatment temperature, the impurity content, particle size and specific surface area decreased, whereas the crystallinity and capacity of the materials increased. When the heat treatment temperature reached 800 °C, the initial Coulombic efficiency of the materials exceeded 90%. The capacity reached approximately 350 mAh/g with a stable cycling performance and almost constant capacity, which is very close to that of CG.

RG exhibited better rate performance than CG, and its capacity did not change during charge–discharge cycling at a low current, indicating its good reversibility Figure 7b The better rate performance of RG can be attributable to the composite structure of the spent anode materials comprising graphite with a layered structure, which was undamaged after long cycling, and amorphous carbon from the pyrolysis of some organic impurities. 

The CV curve of CG and RG is shown in Figure 7d,e. A small peak was observed around 0.65 V for the two materials but disappeared in the subsequent cycle. This peak corresponded to the formation of the SEI layer, which indicates that the SEI layer was removed during this process. The two materials’ integrated areas of the CV curves are close, and the positions of the redox peaks are almost the same, indicating the high purity of RG and its superior performance, which is very close to that of CG. 

Figure 7f indicates that the initial resistance of RG was lower than that of CG. This is because the proposed process did not remove the conductive carbon originally added to the anode materials, and RG contained a small amount of amorphous carbon. All of these various tests indicate that the performance of RG obtained through the proposed process is close to that of CG, with some performance parameters showing better values. Therefore, the proposed method shows extremely high application value and can be directly used for the regeneration of battery materials.

## 4. Conclusions

To achieve high-value recovery of the spent anode materials, they were processed through heat treatment, selective lithium extraction via water immersion and deep impurity removal via acid washing. A lithium leaching solution and RG were obtained through recycling. The optimal heat treatment temperature was determined to be 800 °C, and a protective inert atmosphere was employed during the process. The water immersion solid/liquid ratio was 1:80, and the immersion process was conducted at 25 °C for 30 min. After testing, the leaching ratio of lithium in the spent anode materials reached 87% and there were no other impurities in the leaching solution, which could be directly recycled. RG exhibited high purity, an optimised initial Coulombic efficiency of 90.5% and a capacity of 350.2 mAh/g, with a stable cycling performance and a rate performance superior to that of CG. Thus, the obtained RG can be directly used as a battery-grade graphite material. This study proposes a new, green, simple and efficient method for recycling spent anode materials and maintaining all of their components, considerably increasing their application value.

## Figures and Tables

**Table 1:** Specific surface areas of spent anode materials after heat treatment.

Materials	Specific Surface Area (m^2^/g)
RAW	7.059
CG	1.505
Heat treatment at 600 °C	3.758
Heat treatment at 700 °C	3.285
Heat treatment at 800 °C	3.199
Recycled graphite products	2.027

**Table 2:** Initial Coulombic efficiency and cycle performance of recycled anode materials (600 °C, 700 °C and RG refer to recycled anode materials after heat treatment at 600 °C, 700 °C and 800 °C, respectively).

Materials	Initial Discharge Capacity (mAh/g)	Initial Charge Capacity (mAh/g)	Initial Coulombic Efficiency (%)	50th Charge Capacity (mAh/g)
RAW	362	282.5	78	275
CG	381.4	348	91.2	358
600 °C	338.8	283.9	83.7	331
700 °C	383.6	339.6	88.5	335
RG	386.8	350.2	90.5	349

## Data Availability

The data presented in this study are available on request from the corresponding author.
